# Supplementary material for: Validation of measurement‐guided 3D VMAT dose reconstruction on a heterogeneous anthropomorphic phantom
Source: J Appl Clin Med Phys. 2013 Jul 8;14(4):70–84. doi: 10.1120/jacmp.v14i4.4154 (PMC5714524; doi:10.1120/jacmp.v14i4.4154)
Supplement: Supplementary file 1 — Supplementary Material [file ACM2-14-070-s001.docx]

**Validation of measurement-guided 3D VMAT dose reconstruction on a heterogeneous anthropomorphic phantom**

**Daniel Opp**

*Department of Radiation Oncology, Moffitt Cancer Center, Tampa, Florida 33612*

**Benjamin E. Nelms**

*Canis Lupus LLC, Merrimac, Wisconsin 53561*

**Geoffrey Zhang, Craig Stevens and Vladimir Feygelman**

*Department of Radiation Oncology, Moffitt Cancer Center, Tampa, Florida 33612*

[vladimir.feygelman@moffitt.org](mailto:vladimir.feygelman@moffitt.org)

Running title: Validation of measurement-guided dose reconstruction

**Abstract:** 3DVH software (Sun Nuclear Corp., Melbourne, FL) is capable of generating a volumetric *patient* VMAT dose by applying a volumetric perturbation algorithm based on comparing measurement-guided dose reconstruction and TPS calculated dose to a cylindrical *phantom*. The primary purpose of this paper is to validate this dose reconstruction on an anthropomorphic heterogeneous thoracic phantom by direct comparison to independent measurements. The dosimetric insert to the phantom is novel, and thus the secondary goal is to demonstrate how it can be used for the hidden target end-to-end testing of VMAT treatments in lung.

A dosimetric insert contains a 4 cm diameter unit-density spherical target located inside the right lung (0.21 g/cm^3^ density). It has 26 slots arranged in two orthogonal directions, milled to hold optically stimulated luminescent dosimeters (OSLDs). Dose profiles in three cardinal orthogonal directions were obtained for five VMAT plans with varying degrees of modulation.

After appropriate OSLD corrections were applied, 3DVH measurement-guided VMAT dose reconstruction agreed 100% with the measurements in the unit density target sphere at 3%/3mm level (composite analysis) for all profile points for the 4 less modulated VMAT plans, and for 96% of the points in the highly modulated C-shape plan (from TG-119). For this latter plan, while 3DVH shows acceptable agreement with independent measurements in the unit density target, in the lung disagreement with experiment is relatively high for both the TPS calculation and 3DVH reconstruction. For the 4 plans excluding the C-shape, 3%/3mm overall composite analysis passing rates for 3DVH against independent measurement ranged from 93 to 100%. The C-shape plan was deliberately chosen as a stress test of the algorithm. The dosimetric spatial alignment hidden target test demonstrated the average distance to agreement between the measured and TPS profiles in the steep dose gradient area at the edge of the 2 cm target to be 1.0±0.7, 0.3±0.3, , and 0.3±0.3 mm for the IEC X, Y, and Z directions, respectively.

**Key words:** VMAT QA, Patient dose reconstruction, Treatment verification, Intensity modulation, Phantoms

**PACS:**  87.55Qr

1. **INTRODUCTION**

Historically, it was expected that every inversely-planned radiotherapy course would receive some level of experimental dosimetric quality assurance (QA) prior to first treatment.^(^[^1^](#_ENREF_1)^)^ In the United States, such “patient-specific end-to-end tests” ^(^[^2^](#_ENREF_2)^)^ are currently codified as an essential element of an IMRT safety and QA program ^(^[^3^](#_ENREF_3)^)^ and are a requirement for the facility accreditation in Radiation Oncology by the American College of Radiology.^(^[^2^](#_ENREF_2)^)^ Electronic arrays, either planar ^(^[^4-6^](#_ENREF_4)^)^ or quasi-three-dimensional,^(^[^7-10^](#_ENREF_7)^)^ are a popular practical choice for these tests because of the ease of setup and nearly instantaneous availability of results. Various combinations of dose-error and distance to agreement (DTA) thresholds are used to create comparison metrics between the measured and treatment planning system (TPS) calculated dose on the phantom, either sequentially in the composite analysis approach, ^(^[^11^](#_ENREF_11)^)^ or often combined in the gamma-analysis formalism introduced by Low et al.^(^[^12^](#_ENREF_12)^)^ The analysis passing rates are presumed to reflect the TPS ability to accurately calculate the phantom dose and the method is commonly used during commissioning of a TPS or delivery system. It can catch certain (but not all) types of the larger deviations during the pre-treatment QA. However, regarding the ability to identify the potentially clinically significant but modest dosimetric imperfections in the IMRT process on a per-patient basis ^(^[^13^](#_ENREF_13)^),(^[^14^](#_ENREF_14)^)^ in terms of sensitivity to systematic errors, ^(^[^13-16^](#_ENREF_13)^)^ the value of this metric is less clear. One way to circumvent limitations in the sensitivity of conventional passing rate metrics is to reconstruct full, high-resolution volumetric (3D) patient dose from relatively sparse array measurements, and generate delivered patient-based dose volume histograms (DVHs) for direct comparison with the plan.^(^[^13^](#_ENREF_13)^)^ It was recently demonstrated with independent dosimeters that on a homogeneous “patient” this measurement-guided dose reconstruction is sufficiently accurate for fixed gantry step-and-shoot IMRT with a planar diode array,^(^[^17^](#_ENREF_17)^)^ and for VMAT with a helical one.^(^[^18^](#_ENREF_18)^)^ The goal of this paper is to demonstrate that the latter approach is also valid for a heterogeneous “patient” dataset. To that end, we devised a novel thoracic phantom insert accommodating an ion chamber or an array of optically stimulated luminescent dosimeters (OSLDs) in and around a unit-density spherical target suspended inside the lung-density material. In the process, we also illustrate how this phantom can be used, with a modest effort, for end-to-end hidden target tests.

1. **MATERIALS AND METHODS**
   1. **Phantoms**

The anthropomorphic phantom used in this work is a modification of the commercial IMRT Thorax Phantom (Model 002LFC, CIRS Inc., Norfolk, VA).^(^[^19^](#_ENREF_19)^)^ The conceptual design of the new dosimetric insert was provided by the authors, while the detailed design and manufacturing were performed by the vendor. The phantom is based on a Plastic Water cylinder (1 in Figure 1) with an approximately elliptical cross section flattened on the bottom to provide stability on the table. The overall dimensions are 30 cm x 30 cm x 20 cm. The phantom contains two cylindrical “lungs” (2) made out of epoxy resin 0.21 g/cm^3^ in density. The right lung (appearing with a cavity in Figure 1) can accommodate an 8.5 cm diameter cylindrical lung density insert. One version of the insert contains a 4 cm diameter spherical Plastic Water target with a cavity for an A1SL 0.05 cm^3^ ion chamber (Standard Imaging Inc., Middleton, WI) in the center. The other version (5) is overall geometrically equivalent but has a removable two-piece (3 and 4 in Figure 1, and Figure 2) target insert designed to accommodate optically stimulated luminescent dosimeters (OSLD) (6) known as InLight nanoDots (Landauer Inc., Glenwood, IL). The nanoDot slots are placed such that the lines drawn through the centers of the active volumes form two orthogonal profiles through the target center. The first array (IEC X-direction on the transverse cut in Figure 2) has 14 slots offset symmetrically from the target center. The two central nanoDots are offset ±2.5 mm while the remaining detectors are separated by 5 mm in the target and 4.1 mm in the lung. The orthogonal (IEC Z) set contains 12 detectors positioned asymmetrically with respect to the target center. The innermost dosimeters are shifted +6.5 and -8.5 mm from the center, with the remaining ones following the same spacing pattern as described above. The reduced distance between the outer detectors (4.1 mm) is an attempt to balance the desire for higher detector density in the penumbra region with the mechanical strength of the low density epoxy material. Each detector set covers about 60 mm in length. The “rounded cube” target insert is symmetrical on the outside and the detector plane can be positioned in the cylinder to coincide with any of the cardinal anatomical planes. In addition, the cylinder can be rotated to place the detector plane in any number of intermediate orientations, although only the three cardinal positions were used in this work. The phantom also has a 4 cm diameter “spine” bone density (1.6 g/cm^3^) insert (7 in Figure 1) drilled for an ion chamber.

In addition, for the OSLD sensitivity measurements described below, a 20x20x20 cm^3^ Plastic Water Cube phantom (CIRS Inc., Norfolk, VA) was used. The phantom could interchangeably accommodate either a Semiflex 0.125 cc ion chamber (PTW, Freiburg, Germany) or a single nanoDot.

- 1. **OSL Dosimeters**

The OSL Carbon-doped Aluminum Oxide (Al_2_O_3_:C) dosimeters were originally proposed for radiation protection application in the late 1990’s by Akselrod’s group ^(^[^20^](#_ENREF_20)^)^ and were later characterized for radiotherapy applications by the same ^(^[^21^](#_ENREF_21)^)^ and other ^(^[^22-26^](#_ENREF_22)^)^ investigators. The electrons trapped in the metastable states in the forbidden band as a result of irradiation are liberated by optical stimulation. The resulting photons are counted by a photomultiplier, and the total count is related to absorbed dose through direct calibration. The active element is a 5 mm diameter disk, approximately 0.2 mm thick. It is surrounded by a black plastic casing approximately 10x10 mm^2^ in cross-section and 1 mm thick on each side. Exposed dosimeters are read out by a MicroStar device (Landauer Inc.) with a continuous stimulation LED light source. OSLDs can be reused by applying an optical bleaching cycle.^(^[^23^](#_ENREF_23)^)^

- - 1. **Absolute calibration**

All calibration irradiations were done in a 10x10 cm^2^ 6MV beam at 10 cm depth in a Plastic Water (CIRS Inc.) phantom and 100 cm source to OSLD distance. A 5 mm-thick flexible bolus was included immediately above the nanoDots to prevent air pockets and mechanical damage. OSLDs were intended for repeated use and/or individual calibration, and therefore would undergo at least one optical bleaching cycle ^(^[^23^](#_ENREF_23)^)^ prior to the first useful measurement. It was shown previously that the background signal after optical bleaching is higher, albeit marginally, than for the never irradiated OSLD.^(^[^23^](#_ENREF_23)^)^ Therefore, all dosimeters were pre-irradiated to 200 cGy. Optical bleaching was achieved by exposing them for 12 h to a 17W desktop fluorescence light tube positioned approximately 10 cm away. After that, an initial calibration was performed according to the vendor’s procedure to allow the nanodots to be read out. This group calibration factor was further refined for every individual dosimeter by exposing it to a single known dose and taking a ratio of the observed and expected dose readings. This individual sensitivity correction has to be calculated and applied in a spreadsheet outside of the commercial software. The known calibration dose was chosen to be close to the expected single-exposure dose, as described below for the individual experiments. The detectors were divided into three groups. Seventy-eight nanodots were designated for the centerpiece VMAT measurements. The calibration dose was 200 cGy, as it was for 10 dosimeters designated for the side study of sensitivity change with dose accumulated through repeated irradiation/bleaching cycles. The remaining 36 detectors were used for refinement of the angular corrections and were calibrated at either 200 or 50 cGy. All read-outs were performed at least 30 min after irradiation to allow the transient signal to subside.^(^[^23^](#_ENREF_23)^)^ Throughout this work, each OSLD was read out four times with the results averaged.

- - 1. **Accumulated dose dependence**

The maximum total dose for the VMAT nanoDots, after repeated irradiations, was expected to be around 16 Gy. Our preliminary data indicated that some sensitivity changes may have been happening at that level, prompting us to carry out the sensitivity vs. accumulated dose study. Ten OSLDs were irradiated in increments of 2 Gy to a maximum of 16 Gy. In this, and all other experiments, the MicroStar readout was corrected for each nanoDot by the individual sensitivity factor. The readings were plotted against the known cumulative dose and a second-order polynomial best-fit interpolation curve was produced. A sensitivity correction was applied during each VMAT measurements, based on that curve with the known cumulative historical dose of the nanoDot as an argument.

- - 1. **Angular dependence**

There are somewhat conflicting reports in the literature regarding the possible OSLD’s angular sensitivity dependence.^(^[^22^](#_ENREF_22)^,^[^24^](#_ENREF_24)^)^ While Jursinic ^(^[^22^](#_ENREF_22)^)^ found no dependence of OSLD sensitivity on the angle of incidence, Kerns et al ^(^[^24^](#_ENREF_24)^)^ reported approximately 3.5% sensitivity drop (6MV x rays) for the edge-on irradiation, compared to the beam being perpendicular to the nanoDot plane (calibration geometry). Since this work is concerned with arc treatments, we were more interested in average (if any) angular corrections for full arcs then in sensitivity values at the specific, realistically sparse, individual angles. A 360º arc with a 10x10 cm^2^ jaws opening was planned to deliver 200 cGy at the center of the spherical target in the Thorax Phantom. The phantom was aligned using cone-beam CT (CBCT) with the region of interest centered on the target. The exact dose at the target center was determined with an A1SL ion chamber cross-calibrated in a flat Plastic Water phantom. Two different orientations of the OSLDs were encountered in this work. For Anterior-Posterior (AP, or IEC Z) and Left-Right (LR, or IEC X) profiles, during the full gantry rotation the beam incidence angle changes continuously back and forth from perpendicular to parallel to the dosimeter plane. We will call this the standard orientation. For the Superior-Inferior (SI, or IEC Y), the beam always enters the nanoDot edge-on, but the entrance side changes with gantry rotation. The OSLD active volume center is positioned away from the center of the square casing. The casing design is also asymmetric because of the hinge mechanism that exposes the active element. Two different experiments were performed, with both possible geometries evaluated. NanoDots were inserted into the central slots shifted ±2.5 mm from the center and irradiated two at a time. Four OSLDs were irradiated with their planes in coronal, and 4 in sagittal orientation. The average of these 8 readings represented the dose value for the standard orientation. Eight additional nanoDots were irradiated edge-on (detector plane parallel to the transverse plane). The ratio of the ion chamber dose to the average nanoDot dose for each irradiation geometry constitutes a respective multiplicative angular sensitivity correction factor.

Another consideration is a possible change in sensitivity for the edge-on geometry with distance from the central axis. At 30 mm from the central axis, the incidence ray angle deviates from 90º with respect to the normal to the nanoDot plane by 1.7º. A Plastic Water Cube phantom with a Semiflex 0.125 cc ion chamber was used. The phantom was moved through 5 positions with the chamber center being at a depth of 10 cm, 100 cm away from the source, and shifted from 2.5 to 30 mm from the central axis. The chamber readings in a 10x10 cm^2^ field were recorded. Then the chamber was replaced with a nanoDot positioned vertically in a specially milled Plastic Water holder. Four nanodots were irradiated to approximately 50 cGy at each of the five positions. The ratio of the average OSLD dose at any position to the ion chamber reading is the measure of the relative sensitivity dependence on the longitudinal displacement.

- 1. **Major delivery components**

VMAT plans were generated by Pinnacle v. 9.2 TPS (Philips Radiation Oncology. Fitchburg, WI), exported to Mosaiq v. 2.3 information management system (Elekta Impac, Sunnyvale CA) and delivered with a 6MV beam from a TrueBeam linear accelerator (Varian Medical Systems, Palo Alto CA) with a 120-leaf Millennium MLC (5/10 mm leaves). For all experiments involving absolute measurements, the accelerator output was confirmed to be within 0.2% of nominal with a Farmer chamber constancy check.

Coincidence between the megavoltage and kilovoltage isocenters was confirmed by the vendor’s automated procedure to be within 0.5 mm.

- 1. **Dosimetric verification**
     1. **Treatment planning**

The Thorax Phantom was scanned for treatment planning with 1 mm thick slices. We used 5 VMAT plans for measurements in lung. The first one was optimized to deliver 200 cGy to a 2 cm diameter spherical target centered inside the Plastic Water sphere. The second plan was similar, except the optimization target diameter was increased to 7 cm. The smaller target also allows measurement of profiles in the penumbra (80-20%) and thus evaluation of the geometric accuracy of the dose distribution alignment to isocenter. The larger target provides an opportunity to evaluate dose in lung from an essentially open-field arc. The next three plans were not optimized anew but rather copied from our previous work on the homogeneous phantom.^(^[^18^](#_ENREF_18)^)^ They were modeled after TG-119 ^(^[^27^](#_ENREF_27)^)^ Mock Head and Neck (HN), Multi-Target (MT) and C-shape plans. While not necessarily clinically relevant in lung, these plans provided the necessary varying levels of complexity (modulation) to probe the limits of the dose planning/reconstruction accuracy, with the C-shape plan expected to provide the biggest challenge. All plans were calculated on a 2 mm grid with 2º gantry angle increments using the full Collapsed Cone Convolution algorithm.

- - 1. **Dose reconstruction**

The methods for measurement-guided estimates of the 3D patient dose from the phantom data (planned dose perturbation, or PDP) were previously described and empirically validated on the homogeneous phantoms for static gantry IMRT ^(^[^17^](#_ENREF_17)^)^ and VMAT ^(^[^18^](#_ENREF_18)^)^ treatments. We briefly summarize here the basics of the VMAT method used in conjunction with the helical diode array (ArcCHECK, Sun Nuclear Corp., Melbourne, FL), that is called ArcCHECK planned dose perturbation (ACPDP). The calibrated helical ArcCHECK (AC) diode dosimetry array ^(^[^7^](#_ENREF_7)^)^ records in 50 ms intervals the dose delivered by an arc. The gantry motion and planned control points are synchronized with the dosimeter in absolute time through a virtual inclinometer.^(^[^7^](#_ENREF_7)^)^ The dynamic arc beam is discretized into a series of sub-beams. Each sub-beam has a fixed gantry angle but a variable fluence derived from the planned MLC motions during the time period allocated to this sub-beam (typically 0.2-0.4 s for VMAT treatments). For computational efficiency reasons, the sub-beam time duration is higher than the AC sampling rate, but it still provides angular spacing of ~2º, ensuring adequate accuracy of the subsequent calculation. The relative volumetric dose from each sub-beam is calculated on the homogeneous cylindrical virtual phantom by a convolution engine. These relative dose grids are then morphed based on the measured data from the relatively sparse helical diode array and summed up to produce a high density (TPS voxel resolution - 2mm in this work) 3D absolute dose matrix in the cylindrical phantom. For each voxel in the phantom, a ratio is taken of this measurement-guided reconstructed dose to the planned one. The planned dose on the patient is then modified (perturbed) by the corresponding ratios, to arrive at the measurement-guided estimate of the 3D dose, as described by the following perturbation equation for an arbitrary *i^th^* voxel displaced by a radius vector $\boldsymbol{r}_{\boldsymbol{i}}$ from the isocenter:

$D_{ACPDP}^{Pat}\left( \boldsymbol{r}_{\boldsymbol{i}} \right)=D_{TPS}^{Pat}\left( \boldsymbol{r}_{\boldsymbol{i}} \right)\frac{D_{ACPDP}^{Phant}\left( \boldsymbol{r}_{\boldsymbol{i}} \right)}{D_{TPS}^{Phant}\left( \boldsymbol{r}_{\boldsymbol{i}} \right)}$ (1)

where $D_{ACPDP}^{Pat}$ is the patient dose estimated by ACPDP, $D_{TPS}^{Pat}$ is the patient dose calculated by the TPS, and $D_{ACPDP}^{Phant}$ and $D_{TPS}^{Phant}$ are the doses in phantom reconstructed by ACPDP and calculated by the TPS, respectively. Thus the main premise of the algorithm is that the delivered dose to the *patient* can be estimated by adjusting (morphing) the TPS calculated dose on a voxel-by-voxel basis, by a 3D array of correction factors derived from the comparison of full-volume, high resolution reconstructed and calculated doses on a cylindrical *phantom*.

- - 1. **Dosimetric verification**

To establish a baseline quality of dosimetric agreement, the measurement guided 3D reconstructed dose on the Thorax Phantom (“patient”) was compared to the TPS dose for the 5 plans in the lung using gamma analysis.^(^[^12^](#_ENREF_12)^)^ Voxels receiving at least 20% of the maximum dose were included in the 3D gamma calculation. Passing rates with different threshold combinations (3%/3mm and 2%/2mm) and dose-error normalizations (global vs. local) were evaluated (Table 1).

For the point dose ion chamber comparisons, The Thorax Phantom was squared on the room lasers and aligned on the lung target by CBCT. Point doses in the middle of the spherical target (5 plans) and the bone insert (1 plan) were extracted from both the initial TPS calculation and 3DVH dose reconstruction on the Thorax Phantom. They were compared to the A1SL ion chamber measurements.

For the profile measurements in the Thorax phantom, the two orthogonal linear nanoDot arrays (Figure 2) define a measurement plane. Making this plane coincide with two of the three cardinal anatomical planes yields dose profiles in all three primary orthogonal directions: AP, LR, and SI. By adding a third orthogonal measurement plane orientation, the experiment is made symmetrical in a sense that each profile is measured exactly twice. With the insert orientations we chose, the AP and LR profiles happen to use the same set of nanoDot slots (horizontal in Figure 2) for the two measurements, and the readings were simply averaged. Point doses extracted from the TPS and 3DVH for comparison with the measured nanoDot doses coincided with the centers of the OSLDs’ active volumes. In the SI direction, the nanoDot arrays switch between the two insert orientations. Except for the two central dosimeters (±2.5 mm), the nanoDots’ positions are shifted by 1 mm in the room coordinate system between the two arrays if they are aligned along the same axis (Figure 2). The two readings from the nanodots positioned 1 mm apart in the phantom were averaged and compared to the TPS or 3DVH point doses between the dosimeters. This procedure avoided over-weighting the SI direction in the overall analysis by assigning it a double number of points. Since one of the arrays is sparse in the middle (Figure 1, Figure 2), only the readings from one irradiation were used for the ±2.5 mm positions. The ion chamber dose in the center was compared to the average of all 10 available nanoDot readings in the two central positions. In addition to individual calibration factors, all OSLD doses included corrections for historical accumulated dose, and average angular dependence for an appropriate orientation.

The individual nanoDot doses (all irradiated dosimeters included) were compared to the TPS and 3DVH by composite % dose-difference (local normalization)/distance to agreement (DTA) analysis ^(^[^11^](#_ENREF_11)^)^. This method is similar to γ-analysis ^(^[^12^](#_ENREF_12)^)^ but is mathematically more stringent and thus typically produces slightly lower passing rates. Each measurement point is tested against the reference distribution and fails if the dose difference from the reference point at the same location is above the dose-error threshold and the distance to the closest point with the same dose value is above the DTA threshold. Composite analysis is easier to implement for manual calculations, which is why we use it here for the profiles. The data were further subdivided for the measurement points inside the unit density sphere and in the lung.

- - 1. **Dosimetric spatial alignment**

In the regions of the steep dose gradient on the periphery of the 2-cm target the average DTA between the measured nanoDots profiles and calculated TPS profiles was determined. This was done to demonstrate how the new phantom can be used to assess geometric accuracy of the VMAT dose delivery in a hidden target end-to-end test.

1. **RESULTS**
   1. **Accumulated dose dependence and angular corrections**

At 16 Gy, the sensitivity change was almost -7%. Overall, the second order-polynomial fit the measured data with R^2^=0.96. This form of the fitting curve was chosen because the dependence is known to be non-linear, while the second-order polynomial provided the highest R^2^ value among the standard set of regression curves with generally appropriate shape. Ultimately, these corrections were verified by the case-by-case comparisons with the ion chamber presented later in the manuscript.

The average angular corrections for the edge-on and standard irradiation geometries were 1.020±0.008 (1SD) and 1.008±0.004, respectively. The first value is 1.5% lower than reported by Kerns et al^(^[^24^](#_ENREF_24)^)^ and almost splits the difference between the values obtained in that work and by Jursinic.^(^[^22^](#_ENREF_22)^)^ The second value has no direct counterpart in the cited work but our numerical integration of the Figure 5 curve in Ref ^(^[^24^](#_ENREF_24)^)^ yields the value of 1.022, again 1.4% higher than our result.

There was no difference observed in the nanoDots’ edge-on sensitivity as the dosimeters were progressively moved from 2.5 to 30 mm away from the central axis. The average relative ratio of the OSLD to ion chamber readings was 1.000±0.002 (range 0.998-1.004).

- 1. **Dosimetric verification**

The 3DVH reconstructed patient dose is generally close to the TPS calculations. This means that in these cases, the TPS dose does not need to be perturbed much. The lowest γ-analysis passing rate among the 5 plans ranged from 98.3% for the most lenient 3%(global)/3mm thresholds to 90.9% for the most stringent 2%(local)/2mm combination. The lowest passing rates were always associated with the C-shape plan,^(^[^27^](#_ENREF_27)^)^ with disagreement primarily localized in the low dose low density area corresponding to the avoidance structure, as explored in more detail in the following sections.

The ion chamber measurements for the most part agree well with both 3DVH and the TPS (Table 2), with the notable exception of the C-shape plan, where the isocenter is in the low dose (avoidance) region. It must be mentioned that all percentage errors here are normalized locally. If normalized globally as in the TG-119 report,^(^[^27^](#_ENREF_27)^)^ the % error values for the C-shape would be about ¼ of that presented in Table 2. For this plan, 3DVH substantially reduced the difference from the measured value compared to the TPS.

For the OSLD profile measurements, the ratio of the average nanoDot dose from the two innermost detector positions to the ion chamber was 0.999±0.015 (1SD, range 0.976-1.013).

The composite analysis passing rates for all 5 VMAT plans in the lung are given in Table 2, while dose profiles in the SI and LR directions are presented in Figure 3 for the 4 cases. The AP profiles are included in the numerical analysis but omitted from the figures for clarity. For the first four cases, in the Plastic Water unit density target, 3DVH provides very good agreement (100% composite analysis passing rate for the nanoDot points), while the TPS agrees with the measured profiles for 93% of the points in the worst case scenario. The passing rates in the lung are somewhat lower. However the difference between 3DVH and measurement in the low gradient region in lung never exceeded 3.9%. For the last case in the lung – the C-shape–Figure 4A shows a persistent area of disagreement between the TPS and 3DVH in the low dose area (central avoidance structure). In the water-equivalent target 3DVH reconstruction agrees well with the measured dose (Figure 3D, Table 2), unlike the TPS. However the disagreement between the measured dose and 3DVH in lung remains substantial (up to 14% normalized locally, or 10 cGy over 72 cGy), although smaller than with the TPS (up to 28%, Figure 3).

- 1. **Dosimetric spatial alignment**

The average distance to agreement between the measured and TPS profiles in the steep dose gradient area at the edge of the 2 cm target was 0.3±0.3, 1.0±0.7, and 0.3±0.3 mm for AP, LR and SI directions, respectively. This easily demonstrates 2-mm spatial dosimetric accuracy at worst.

1. **DISCUSSION**
   1. **Accumulated dose dependence and angular corrections**

In our experiments, the OSLD sensitivity dropped faster with accumulated dose than reported by Jursinic,^(^[^23^](#_ENREF_23)^)^ who found no sensitivity changes up to 20 Gy. However Kerns et al ^(^[^24^](#_ENREF_24)^)^ quoted accumulated dose of 10 Gy as the threshold beyond which the OSLD sensitivity may change, based on the work of Reft ^(^[^28^](#_ENREF_28)^)^ and Yukihara et al. ^(^[^29^](#_ENREF_29)^)^ In turn, Yukihara et al. ^(^[^29^](#_ENREF_29)^)^ noted a possible inconsistency between the sensitivity drop and supralinearity results reported together.^(^[^22^](#_ENREF_22)^)^ Investigation of these somewhat conflicting results in the literature is beyond the scope of this work. However we should point out certain differences in materials and methods that could potentially be contributing to the observed differences. Kerns et al ^(^[^24^](#_ENREF_24)^)^ noted that older models of the OSLDs and the reader were used by Jursinic.^(^[^23^](#_ENREF_23)^)^ In addition, a bleaching light source with a different spectrum (tungsten-halogen lamp) was used in that work, as opposed to a fluorescent bulb in this paper. The tungsten-halogen light has higher photon energy and is thus more efficient in releasing electrons and holes from the deep traps which are responsible, in a complex fashion, for the OSLD sensitivity dependence on radiation history.^(^[^29^](#_ENREF_29)^)^

But most importantly, in the context of the current paper, the difference from the previously described sensitivity behavior is not a concern, as long as the dosimeters’ behavior is consistent throughout the measurement series. To that end, the average reading of 10 OSLDs was spot-checked against an ion chamber for each plan, and found to be internally consistent with the calibration results.

Assuming for a moment that the only source of error in the angular correction measurements is the statistical variation of the nanoDot readings, the estimated coefficients of variation from Kerns et al ^(^[^24^](#_ENREF_24)^)^ (~1%) and our measurements (0.4%) add up to 1.4%, thus overlapping at the 1 SD level. In reality, the phantoms and irradiation conditions were different and other sources of error exist, so it is not possible to conclude that the results are different. We elected to use our values as directly related to the experimental setup at hand. Kerns et al ^(^[^24^](#_ENREF_24)^)^ reported the maximum angular correction factor to differ by up to 4% from the earlier report,^(^[^22^](#_ENREF_22)^)^ with the reason not determined.

- 1. **Dosimetric verification**
     1. **Dose profiles in lung - nanoDots**

The ratio of the average nanoDot dose from the two innermost detector positions to the ion chamber in the VMAT experiments (0.999) confirms the validity of our overall correction formalism. The largest difference (-2.4%) is associated with the C-shape plan. Given the narrow MLC openings hovering over the measurement point, and a large amount of time it spends under the nominally closed leaves, it is reasonable to expect that the difference in the effective detector shapes (a cylindrical ion chamber volume vs. two thin OSLD disks separated by 5 mm) could contribute to the observed dose difference. Excluding the C-shape plan, the difference between the OSLDs and the ion chamber in the center did not exceed 1.3%.

The lower passing rates in the lung are associated with the plans where there is no sharp dose gradient (7 cm target and HN, Figure 3 A,B). In those plans, the TPS errors in the heterogeneous media are not hidden in the distance to agreement analysis. The largest disagreements in the low gradient area between 3DVH and measurements for the moderately modulated plans do not exceed 3% in the unit density target and 4% in the lung material. Overall, this error magnitude is quite reasonable for a heterogeneous anthropomorphic phantom, particularly considering that all measurement points in the lung are actually in the vicinity (≤ 10 mm) of the interface with the unit density target. The direction of the error coincides with the ion chamber data we collected for a few simple open fields in a flat phantom containing a low density wood heterogeneity.

It must also be mentioned that while we scored the points with dose-difference between 3 and 4% as failing the dose portion of the analysis, the diode array analysis methodology in TG-119 ^(^[^27^](#_ENREF_27)^)^ would have considered them passing, because of the additional 1% “measurement uncertainty” padding included.

Since some plans exhibited passing rates up to 100% with the 3%/3mm threshold combination (Table 2), composite analysis results with more stringent 2%(local normalization)/2mm criteria are presented in Table 3.

One must be cautious in inferring from the Tables 2 or 3 if 3DVH or the TPS has a “better agreement” with the OSLD measurements. As one can see from Table 1, some plans show nearly 100% agreement between the TPS and 3DVH at the 2%(local normalization)/2mm level. Both 3DVH/ArcCHECK, and the OSLD are reasonably accurate in practical terms, but both have measurement uncertainties of the same order of magnitude as the errors being investigated. This underscores the larger overall dilemma with the current IMRT dosimetry techniques.^(^[^30^](#_ENREF_30)^)^ As a result, it is fully expected that when the TPS and 3DVH dose distributions are this close, in any given case one or the other may show a slightly better agreement with another dosimetry system. Thus for the 4 plans that do not include the C-shape, the results of the composite analysis are similar between the TPS and 3DVH, with the passing rate being somewhat higher or lower for either one in any given case, based on the small differences in the profiles (Figure 3). The ultimate goal of this paper is not to demonstrate that 3DVH dose errors vs. an independent dosimetry array are less than with the TPS for every plan, but rather to show that measurement-guided dose reconstruction in a phantom representing a patient is sufficiently accurate by the industry standard, based on the comparison with an independent set of dosimeters.

The last case in the lung – the C-shape– stands out and is an excellent illustration of both the power and limitations of 3DVH. With the points in the C-shape avoidance structure spending substantial amount of time under the closed leaves and being repeatedly exposed to the narrow MLC openings (Figure 4 B-D), it can be assumed that one or both of these factors are responsible for the disagreement between the TPS and independent measurement in the unit density sphere. 3DVH is able to account for these errors in the 3D dose distribution through its measurement-derived correction matrix. However the disagreement between the measured dose and 3DVH in lung remains substantial, albeit smaller than with the TPS (Figure 3D). We cannot definitively prove the cause of this error. In fact, given the challenges of the small-field dosimetry, particularly in the low-density media and with the dynamic MLC delivery, it might be a rather challenging task. However, better agreement between 3DVH and measurements observed previously for the same plan in a fully homogeneous phantom (ion chamber and film) ^(^[^18^](#_ENREF_18)^)^ and in the unit density sphere in this work (nanoDots and ion chamber), point towards the TPS inability to accurately calculate dose in the low density media in the very narrow fields. The Pinnacle Collapsed Cone Convolution algorithm is generally considered reliable in the lung.^(^[^31^](#_ENREF_31)^)^ While, to our knowledge, no study directly described the challenging irradiation geometry close to the C-shape VMAT plan, it has been noted previously that convolution/superposition algorithms are likely to lose accuracy with small fields in the low density media, due to the lack of lateral electronic equilibrium.^(^[^32^](#_ENREF_32)^,^[^33^](#_ENREF_33)^)^ In addition, our measurement points in lung are all within 1 cm of the unit density sphere, making the TPS calculations even more challenging because of the proximity to the interface. The current 3DVH algorithm is based on the homogeneous phantom model and thus by design does not attempt to account for heterogeneity-specific TPS calculation inaccuracies. The C-shape plan served as a good stress test of the 3DVH algorithm in a heterogeneous phantom. Again, all the discussed % dose-errors are locally normalized. If expressed as a percentage of a higher value, typically prescription dose, as is the *de-facto* standard of practice promulgated by the AAPM TG-119 Report,^(^[^27^](#_ENREF_27)^)^ the difference between 3DVH and independent dosimeters for the C-shape plan along the SI profile in lung would be under 5%, which should be considered satisfactory for a low-density avoidance region in a highly modulated plan on an anthropomorphic heterogeneous phantom.

In this work, 3DVH measurement-guided dose reconstruction was confirmed by measurements with independent dosimeters to approximate VMAT dose on a heterogeneous thoracic phantom with acceptable accuracy. Alternative approaches to patient dose reconstruction exist. One commercially available device (COMPASS, IBA Dosimetry) uses a gantry-mounted detector matrix to determine radiation fluence in air that is projected on the CT dataset to approximate patient dose.^(^[^34^](#_ENREF_34)^)^ While good agreement with measurements was reported,^(^[^34^](#_ENREF_34)^)^ the device is yet to be validated with dosimetrically challenging lung test cases.

Another approach to 3D dose reconstruction on a patient involves EPID dosimetry. One version is based on the independent calculation,^(^[^35^](#_ENREF_35)^)^ in its most sophisticated form using a Monte Carlo engine,^(^[^36^](#_ENREF_36)^)^ of the patient dose based on the fluence measured by an EPID in air. The results comparing the dose reconstruction to film dosimetry on a thoracic phantom are encouraging for conventional and IMRT treatments.^(^[^37^](#_ENREF_37)^)^ However, this approach was characterized as rather complex ^(^[^38^](#_ENREF_38)^)^ and an alternative using exit EPID dosimetry in conjunction with a back projection reconstruction was suggested.^(^[^39^](#_ENREF_39)^)^ In turn, the accuracy of the back projection dose reconstruction for the lung cancer VMAT treatments appears to be inadequate due to water-based scatter correction kernels.^(^[^38^](#_ENREF_38)^)^ Wendling *et al* ^(^[^38^](#_ENREF_38)^)^ reported a workaround whereby a homogeneous dataset was substituted for the heterogeneous patient, which they termed *in aqua vivo* dosimetry. While improving the agreement between the planned and reconstructed doses, and allowing, for example, to detect the shifts in patient position, this appears to defeat the goal of comparing the planned and deliverable 3D dose distributions (DVHs) on the actual patient CT dataset. Like 3DVH, this method by design cannot detect errors specific to the TPS handling of heterogeneities.^(^[^38^](#_ENREF_38)^)^ Finally, to our knowledge no EPID-based method for measurement-guided patient dose reconstruction is currently commercially available.

- - 1. **Dosimetric spatial alignment**

While there are many ways to prove the alignment of the dose distribution to the isocenter in a hidden target end-to-end test, for a department that owns an OSLD reader using the nanoDots may be a viable alternative. Absolute dose profiles can be obtained with relative ease, simultaneously verifying the dose in the center and the lateral fall-off. In this paper, dose profiles’ spatial alignment under 2 mm was easily demonstrated, which is satisfactory compared to the typical lung SBRT CTV to PTV expansion margins (≥5 mm),^(^[^40^](#_ENREF_40)^)^ and the Radiological Physics Center end-to-end thoracic phantom localization reproducibility (2.4 mm) ^(^[^41^](#_ENREF_41)^)^ or distance to agreement threshold (5mm) in their credentialing test.

**V. CONCLUSIONS**

3DVH measurement-guided VMAT dose reconstruction compared favorably with the independent measurements in the unit density mass inside the lung volume of the thoracic phantom. Composite analysis with 3% (local)/3mm thresholds yielded 100% agreement for 4 VMAT plans, and 96% passing rate for the last, rather complex one. Dosimetric agreement in the lung is slightly worse, reflecting the fact that by design 3DVH does not attempt to correct the errors related to the TPS dose calculations in the low-density media. The largest errors are associated with the highly modulated plan, which is useful as a stress test of the algorithm. These errors are primarily localized in the low-dose region corresponding to a central avoidance structure with lung density. Such a combination can be deemed uncommon in a patient. Finally, the novel phantom allows performing an end-to-end hidden target VMAT test in a thoracic phantom with a modest effort. For best results, nanoDot dosimeters should be individually calibrated and average angular correction factors applied.

With the accuracy of 3DVH patient dose reconstruction demonstrated on an anthropomorphic heterogeneous phantom, we can proceed towards the ultimate goal of deliverable dose estimates on 4D datasets.^(^[^18^](#_ENREF_18)^)^

**ACKNOWLEDGEMENTS**

This work was supported in part by a grant from Sun Nuclear Corporation. Cassandra Stambaugh’s help with collecting and organizing the OSLD data is appreciated.

**REFERENCES**

1. Ezzell GA, Galvin JM, Low D et al. Guidance document on delivery, treatment planning, and clinical implementation of IMRT: report of the IMRT Subcommittee of the AAPM Radiation Therapy Committee. Med Phys. 2003; 30 (8):2089-2115.

2. ACR –ASTRO Practice guideline for intensity modulated radiation therapy (IMRT). <http://www.acr.org/~/media/ACR/Documents/PGTS/guidelines/IMRT.pdf> [cited 2012 6/18/2012].

3. Moran JM, Dempsey M, Eisbruch A et al. Safety considerations for IMRT: Executive summary. Practical Radiation Oncology. 2011; 1 (3):190-195.

4. Poppe B, Blechschmidt A, Djouguela A et al. Two-dimensional ionization chamber arrays for IMRT plan verification. Med Phys. 2006; 33 (4):1005-1015.

5. Herzen J, Todorovic M, Cremers F et al. Dosimetric evaluation of a 2D pixel ionization chamber for implementation in clinical routine. Phys Med Biol. 2007; 52 (4):1197-1208.

6. Jursinic PA, Nelms BE. A 2-D diode array and analysis software for verification of intensity modulated radiation therapy delivery. Med Phys. 2003; 30 (5):870-879.

7. Kozelka J, Robinson J, Nelms B et al. Optimizing the accuracy of a helical diode array dosimeter: A comprehensive calibration methodology coupled with a novel virtual inclinometer. Med Phys. 2011; 38 (9):5021-5032.

8. Letourneau D, Publicover J, Kozelka J et al. Novel dosimetric phantom for quality assurance of volumetric modulated arc therapy. Med Phys. 2009; 36 (5):1813-1821.

9. Yan G, Lu B, Kozelka J et al. Calibration of a novel four-dimensional diode array. Med Phys. 2010; 37 (1):108-115.

10. Bedford JL, Lee YK, Wai P et al. Evaluation of the Delta4 phantom for IMRT and VMAT verification. Phys Med Biol. 2009; 54 (9):N167-176.

11. Harms Sr WB, Low DA, Wong JW et al. A software tool for the quantitative evaluation of 3D dose calculation algorithms. Med Phys. 1998; 25 (10):1830-1836.

12. Low DA, Harms WB, Mutic S et al. A technique for the quantitative evaluation of dose distributions. Med Phys. 1998; 25 (5):656-661.

13. Zhen H, Nelms BE, Tomé WA. Moving from gamma passing rates to patient DVH-based QA metrics in pretreatment dose QA. Med Phys. 2011; 38 (10):5477-5489.

14. Nelms BE, Zhen H, Tomé WA. Per-beam, planar IMRT QA passing rates do not predict clinically relevant patient dose errors. Med Phys. 2011; 38 (2):1037-1044.

15. Kruse JJ. On the insensitivity of single field planar dosimetry to IMRT inaccuracies. Med Phys. 2010; 37 (6):2516-2524.

16. Yan G, Liu C, Simon TA et al. On the sensitivity of patient-specific IMRT QA to MLC positioning errors. J Appl Clin Med Phys. 2009; 10 (1):2915.

17. Olch AJ. Evaluation of the accuracy of 3DVH software estimates of dose to virtual ion chamber and film in composite IMRT QA. Med Phys. 2012; 39 (1):81-86.

18. Nelms BE, Opp D, Robinson J et al. VMAT QA: Measurement-guided 4D dose reconstruction on a patient. Med Phys. 2012; 39 (7):4228-4238.

19. Solberg TD, Medin PM, Mullins J et al. Quality Assurance of Immobilization and Target Localization Systems for Frameless Stereotactic Cranial and Extracranial Hypofractionated Radiotherapy. Int J Radiat Oncol Biol Phys. 2008; 71 (1):S131-S135.

20. McKeever SWS, Akselrod MS. Radiation dosimetry using pulsed optically stimulated luminescence of Al2O3 :C. Radiat Prot Dosim. 1999; 84:317-320.

21. Gaza R, McKeever SW, Akselrod MS. Near-real-time radiotherapy dosimetry using optically stimulated luminescence of Al2O3:C: mathematical models and preliminary results. Med Phys. 2005; 32 (4):1094-1102.

22. Jursinic PA. Characterization of optically stimulated luminescent dosimeters, OSLDs, for clinical dosimetric measurements. Med Phys. 2007; 34 (12):4594-4604.

23. Jursinic PA. Changes in optically stimulated luminescent dosimeter (OSLD) dosimetric characteristics with accumulated dose. Med Phys. 2010; 37 (1):132.

24. Kerns JR, Kry SF, Sahoo N et al. Angular dependence of the nanoDot OSL dosimeter. Med Phys. 2011; 38 (7):3955.

25. Viamonte A, da Rosa LAR, Buckley LA et al. Radiotherapy dosimetry using a commercial OSL system. Med Phys. 2008; 35 (4):1261.

26. Yukihara EG, Mardirossian G, Mirzasadeghi M et al. Evaluation of A_2_O_3_:C optically stimulated luminescence (OSL) dosimeters for passive dosimetry of high-energy photon and electron beams in radiotherapy. Med Phys. 2008; 35 (1):260.

27. Ezzell GA, Burmeister JW, Dogan N et al. IMRT commissioning: multiple institution planning and dosimetry comparisons, a report from AAPM Task Group 119. Med Phys. 2009; 36 (11):5359-5373.

28. Reft CS. The energy dependence and dose response of a commercial optically stimulated luminescent detector for kilovoltage photon, megavoltage photon, and electron, proton, and carbon beams. Med Phys. 2009; 36 (5):1690.

29. Yukihara EG, McKeever SWS. Optically stimulated luminescence (OSL) dosimetry in medicine. Phys Med Biol. 2008; 53 (20):R351-R379.

30. Gordon JJ, Gardner JK, Wang S et al. Reliable detection of fluence anomalies in EPID-based IMRT pretreatment quality assurance using pixel intensity deviations. Med Phys. 2012; 39 (8):4959-4975.

31. Papanikolaou N, Battista JJ, Boyer AL et al. Tissue inhomogeneity corrections for megavoltage photon beams: Report of Task Group No. 65 of the Radiation Therapy Committee: AAPM report No.85. Madison: Medical Physics Publishing; 2004. 135 p.

32. Wachowicz K, Stanescu T, Thomas SD et al. Implications of tissue magnetic susceptibility-related distortion on the rotating magnet in an MR-linac design. Med Phys. 2010; 37 (4):1714-1721.

33. Woo MK, J.R.Cunningham. The validity of the density scaling method in primary electron transport for photon and electron beams. Med Phys. 1990; 17 (2):187-194.

34. Boggula R, Lorenz F, Mueller L et al. Experimental validation of a commercial 3D dose verification system for intensity-modulated arc therapies. Phys Med Biol. 2010; 55:5619-5633.

35. Renner WD, Norton K, Holmes T. A method for deconvolution of integrated electronic portal images to obtain incident fluence for dose reconstruction. J Appl Clin Med Phys. 2005; 6 (4):22-39.

36. van Elmpt W, Nijsten SMJJG, Dekker ALAJ et al. A Monte Carlo based three-dimensional dose reconstruction method derived from portal dose images. Med Phys. 2007; 33 (7):2426-2434.

37. van Elmpt W, Nijsten SMJJG, Dekker ALAJ et al. Treatment verification in the presence of inhomogeneities using EPID-based three-dimensional dose reconstruction. Med Phys. 2007; 34 (7):2816-2826.

38. Wendling M, McDermott LN, Mans A et al. In aqua vivo EPID dosimetry. Med Phys. 2012; 39 (1):367-377.

39. Wendling M, McDermott LN, Mans A et al. A simple backprojection algorithm for 3D in vivo EPID dosimetry of IMRT treatments. Med Phys. 2009; 36 (7):3310-3321.

40. Benedict SH, Yenice KM, Followill D et al. Stereotactic body radiation therapy: the report of AAPM Task Group 101. Med Phys. 2010; 37 (8):4078-4101.

41. Followill DS, Evans DR, Cherry C et al. Design, development, and implementation of the radiological physics center's pelvis and thorax anthropomorphic quality assurance phantoms. Med Phys. 2007; 34 (6):2070-2076.

^(^[^18^](#_ENREF_18)^)^

TABLES

Table 1. Gamma analysis passing rates: 3DVH dose reconstruction on the Thoracic phantom vs. the TPS calculated dose. 3D γ-analysis with the low dose threshold 20%was used. The passing rates are presented with both Global (maximum) and Local dose-error normalization.

| Plan | 3DVH vs. TPS γ-analysis passing rates (%) | | | |
| --- | --- | --- | --- | --- |
|  | 3%/3mm Global | 3%/3mm Local | 2%/2mm Global | 2%/2mm Local |
| 2 cm | 100 | 99.8 | 100 | 99.6 |
| 7 cm | 100 | 100 | 100 | 100 |
| H&N | 99.6 | 96.4 | 97.8 | 92 |
| Multi-Target | 99.8 | 97.7 | 97.8 | 94.9 |
| C-shape | 98.3 | 94.3 | 95.8 | 90.9 |

Table 2. Percent difference in point doses on the Thorax Phantom: measurement-guided dose reconstruction (3DVH) and TPS vs. the ion chamber (IC), and composite analysis (OSLDs) passing rates overall (All), in the unit density central sphere (Water), and Lung. 3%/3 mm thresholds with Local % dose-error normalization were used.

|  | 3DVH | | | | TPS | | | |
| --- | --- | --- | --- | --- | --- | --- | --- | --- |
|  | 3DVH – IC (%) | Composite analysis pass rate 3%/3mm (%) | | | TPS – IC (%) | Composite analysis pass rate 3%/3mm (%) | | |
|  |  | All | Water | Lung |  | All | Water | Lung |
| 2 cm | 0.0 | 100 | 100 | 100 | 0.8 | 98 | 96 | 94 |
| 7 cm | -0.2 | 93 | 100 | 83 | -0.1 | 98 | 100 | 94 |
| H&N | 1.0 | 93 | 100 | 83 | -1.8 | 88 | 93 | 78 |
| Multi-Target | -1.9 | 100 | 100 | 100 | -1.6 | 98 | 100 | 94 |
| C-shape | 4.9 | 85 | 96 | 72 | -12.5 | 59 | 48 | 72 |
| Bone | 1.5 |  |  |  | 2.6 |  |  |  |

Table 3. Measurement-guided dose reconstruction (3DVH) vs. TPS composite analysis passing rates overall (All), in the unit density central sphere (Water), and Lung. 2%/2 mm thresholds with Local % dose-error normalization were used.

|  | 3DVH | | | TPS | | |
| --- | --- | --- | --- | --- | --- | --- |
|  | Composite analysis pass rate 2%/2mm (%) | | | Composite analysis pass rate 2%/2mm (%) | | |
|  | All | Water | Lung | All | Water | Lung |
| 2 cm | 90 | 96 | 83 | 88 | 83 | 94 |
| 7 cm | 68 | 83 | 50 | 88 | 96 | 78 |
| H&N | 88 | 96 | 78 | 76 | 87 | 61 |
| Multi-Target | 98 | 96 | 100 | 98 | 100 | 94 |
| C-shape | 78 | 91 | 61 | 32 | 30 | 33 |

**FIGURE CAPTIONS**

Figure 1. An inferior view of the Thoracic phantom. 1: Plastic Water elliptical body. 2: “Lungs” (~0.21 g/cc). 3 and 4: Lung material dosimetric 2-piece insert with the Plastic Water target and Nanodot slots. 5: A two-piece lung cylinder accommodating the dosimetric insert in different orthogonal orientations. 6: A nanoDot. 7: Bone-density insert.

Figure 2. NanoDot dosimetry insert cross-section in one of the transverse orientations. Highlighted are the spherical target, “rounded cube” lung-equivalent target housing, and nanoDot slots. All dimensions are in mm.

Figure 3. AP (left axes) and SI (right axes) dose profiles for 7-cm target (A), Mock Head and Neck (B), Multi-Target (C), and C-shape (D) VMAT plans. Pinnacle calculations (TPS), 3DVH measurement-guided reconstruction (3DVH) and independent measurements (OSLD + ion chamber in the center) are compared. The left and right scales are shifted to separate the profiles for better visualization. Otherwise AP and SI profiles would intersect at zero distance from the center. The central ±20 mm of every profile is through the unit-density sphere, while the rest is through the lung material.

Figure 4. A: Dose distribution on the coronal slice through the center indicating the area of disagreement between the TPS and 3DVH for a C-shape plan on the Thorax Phantom at the 5% (local) /3mm level. B-D: three consecutive control points from the C-shape plan with narrow MLC openings crossing the center line.
